# Successful conservative treatment for left ventricular free wall rupture after acute myocardial infarction

**DOI:** 10.1186/s13019-023-02397-w

**Published:** 2023-10-07

**Authors:** Haruyuki Kinoshita, Munehiro Kanegawa, Masashi Morita, Shiori Maeda, Yoji Sumimoto, Kenji Masada, Takashi Shimonaga, Toshifumi Hiraoka, Katsuhiko Imai, Hiroshi Sugino

**Affiliations:** 1 Department of Cardiology, National Hospital Organization Kure Medical Center and Chugoku Cancer Center, Aoyamacho 3-1, Kure, 737-0023 Japan; 2 Department of Cardiovascular Surgery, National Hospital Organization Kure Medical Center and Chugoku Cancer Center, Aoyamacho 3-1, Kure, 737-0023 Japan

**Keywords:** Left ventricular free wall rupture (LVFWR), Acute myocardial infarction (AMI), Conservative treatment, Cardiac tamponade, Cardiogenic shock

## Abstract

Left ventricular free wall rupture (LVFWR) is a rare but fatal complication of acute myocardial infarction (AMI). An 81-year-old female patient with several cardiovascular risk factors presented to the emergency department with symptoms of developing a chronic stomachache and cold sweat. An echocardiograph showed wall motion abnormalities from the lateral to posterior wall, as well as pericardial effusion containing clots of up to 17 mm in the posterior wall that indicated LVFWR after AMI. Although she was conscious after being brought to the initial care unit, she suddenly lost consciousness and fell into electromechanical dissociation (EMD). Endotracheal intubation was immediately initiated and her pericardial drainage and intra aortic balloon pump (IABP) placement, and hemodynamics recovered. Although she had 100% obstruction in the left circumflex artery (LCX) #12 on coronary angiography (CAG), she was discharged to the Intensive Care Unit (ICU) without percutaneous coronary intervention (PCI). Conservative treatment such as intubation, sedation, pericardiocentesis and strict blood pressure management as well as treatment by IABP long-term support led to the patient being uneventfully discharged after 60 days.

## Background

Mechanical complications of acute myocardial infarction (AMI) often cause serious failures in systemic circulation, such as left ventricular free wall rupture (LVFWR), ventricular septal perforation (VSP), and papillary muscle rupture (PMR); among which LVFWR is the most serious. LVFWR results in 0.8 to 6.2% of AMI complications [1, 2]. Once this disease has broken out, it accounts for 20% of the cause of death for AMI [2, 3]. The interval between outset to death is short. Therefore, determination of early surgery is necessary, however, the prognosis continues to be severe [4]. Various strategies for surgical repair have been previously reported. However, there are still few reports of patients whose hemodynamics improved with conservative treatment alone without surgical intervention, and suitable cases and optimal management are still unclear. Recently, we encountered a patient who was medically managed and saved from LVFWR after AMI.

## Case presentation

An 81-year-old woman with no prior history of myocardial infarction was emergently transported to our hospital after developing a chronic stomachache and cold sweat. According to her medical history, her current medications included 1 mg of prednisolone and 4 mg of methotrexate to treat lipid abnormalities and rheumatoid arthritis.

**Fig. 1:**
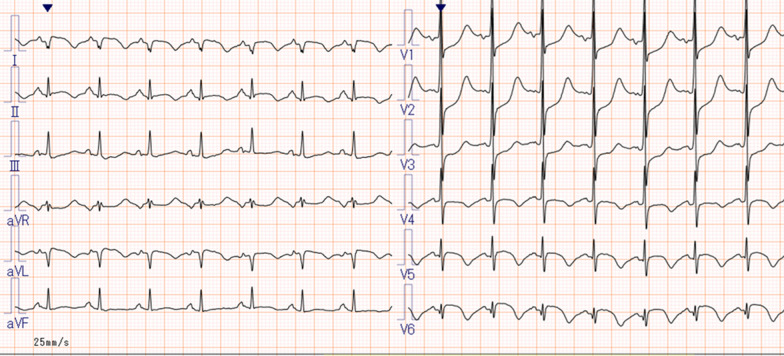
12-lead ECG. HR 111/min regular, I aVL V5–V6 ST elevation, V1–V3 ST depression, III V4–V6 negative T wave, suggesting posterior wall STEMI

**Fig. 2:**
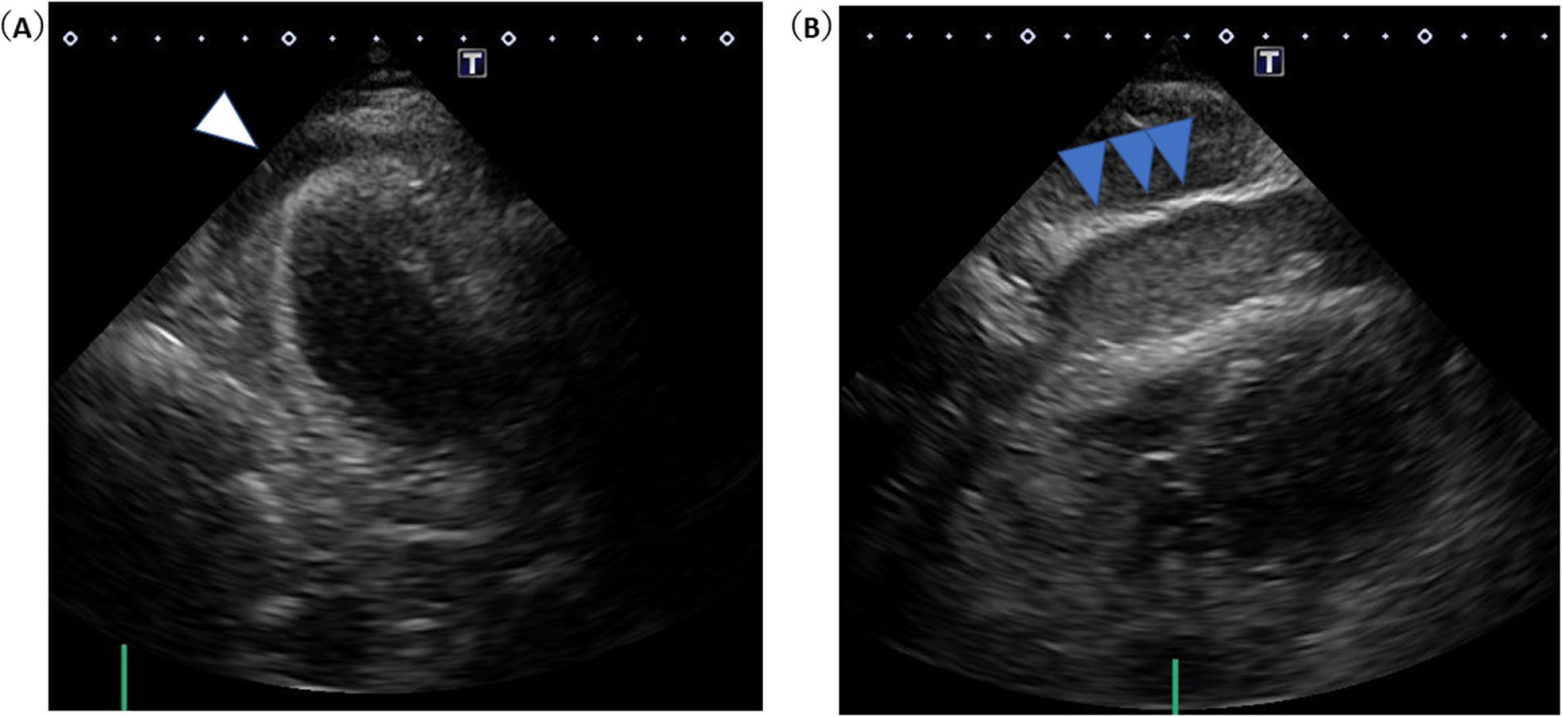
Bedside echocardiography (immediately after transportation). Bedside echocardiography immediately after transfer (**A**) showed an echo-free space of approximately 5–8 mm, suggesting a clot around the posterior wall (triangle). Twenty-five minutes later (**B**), a high bright echo-free space was observed that rapidly expanded to more than 30 mm in the anterior right ventricle (triangle).

**Fig. 3:**
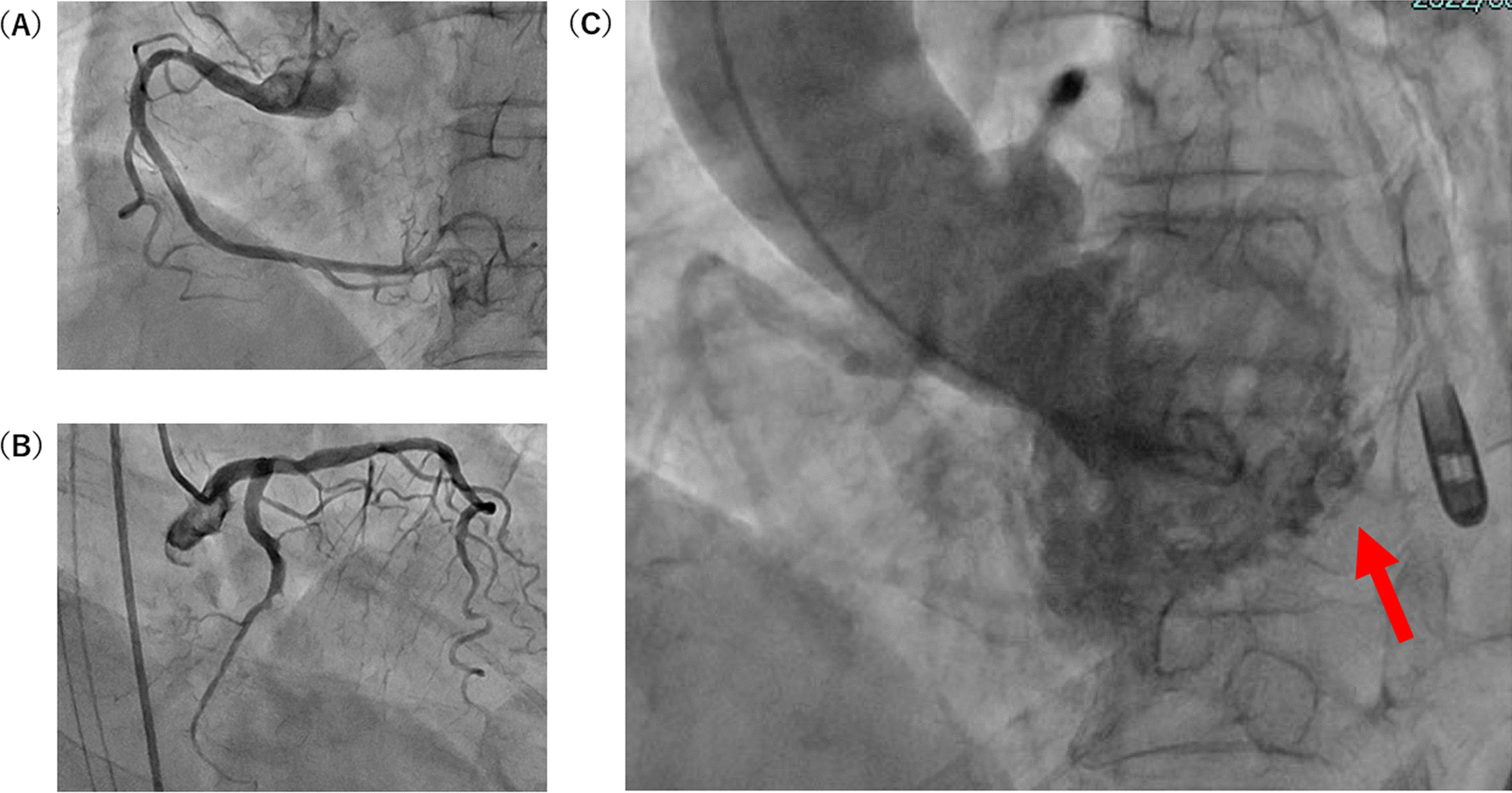
Coronary angiography and left ventriculography. No stenosis was observed in the right coronary artery, and 100% occlusion was observed in the left circumflex coronary artery just after the bifurcation of the obtuse marginal branch (**A**) (**B**). In the LVG, LAO view confirmed the oozing of the contrast agent into the pericardium at the posterior wall region (**C)** (arrow)

**Fig. 4:**
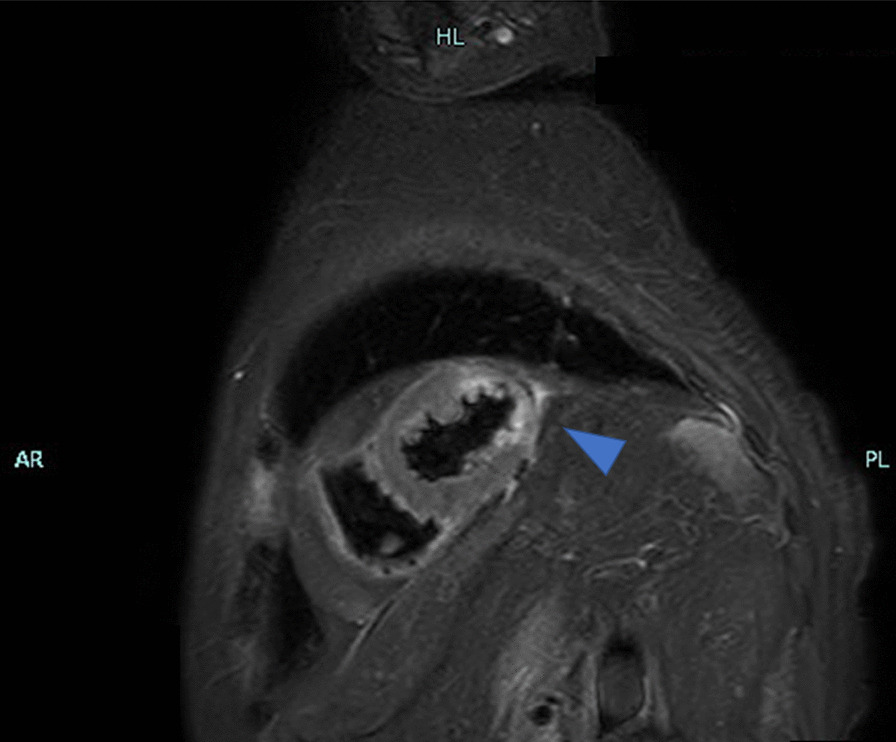
Contrast-enhanced MRI image on the 33. T2WI showed high signal mainly subintima (partially penetrating) from the lateral wall to the posterior wall, which coincides with the posterior aspect of the heart (triangle)

Her vital signs were: Glasgow Coma Scale (GCS) E3V4M6 13 points, pupil 3.5/3.5 mm+/+, body temperature 35.8 °C, heart rate 121 beats/min, breathing 24 times/min, and an undetectable blood pressure. Electrocardiogram (ECG) detected a sinus rhythm, right axis deviation, ST elevation in I, aVL, V5–V6, and an inverted T wave. There was V1–V3-led reciprocal change (Fig. 1) with AMI. She was recognized as having an increase in CK (Creatine Phosphorus Kinase), CK-MB (Creatine Phosphorus Kinase-MB) and cTnI (Cardiac Troponin I) (Table 1) and was diagnosed as ST elevation myocardial infarction (STEMI). Echocardiography showed abnormal wall motion from the lateral to the posterior wall, but the ejection fraction (EF) was around 50% and the cardiac function was relatively preserved. There was no observed valve dysfunction such as mitral regurgitation or aortic regurgitation, or ventricular septal shunt blood flow. Pericardial effusion containing a maximum 17 mm thrombus was observed in the posterior wall, and the sudden increase caused collapse of the right ventricle and deterioration of left ventricular function (Fig. 2). She was diagnosed with AMI and cardiogenic shock due to a cardiac rupture. Adrenaline and noradrenaline were administered to maintain her blood pressure, but her level of consciousness suddenly dropped. After intubation, she was taken to the catheter laboratory and pericardial drainage was performed, and 200 ml of bloody effluent was collected. The pulse became palpable and an intra aortic balloon pump (IABP) was immediately inserted through the right femoral artery. The patient underwent a coronary angiograph, which revealed 100% occlusion of the left circumflex #12 with thrombus. The patient had no significant findings in all other coronary arteries and no collateral circulation. The left ventricular angiograph (LVG) showed oozing of the posterior wall into the epicardium consistent with the heartbeat (Fig. 3). Considering the possibility of re-bleeding due to heparin use, percutaneous coronary intervention (PCI) was not performed. At a heart team conference including cardiovascular surgeons, emergency surgery was considered, but the patient was elderly, hemodynamically stable after admission to the Intensive Care Unit (ICU), and surgical operation on fragile myocardial tissue was judged to carry a high risk of hemostasis difficulty and transition to a blow-out type. Surgical intervention was initiated when re-rupture or progression of pericardial effusion was observed during conservative treatment. The patient continued to maintain her circulatory status, and blood pressure was strictly controlled in a range of 80–100 mmHg to prevent re-rupture, and no aspirin or heparin antithrombotic or anticoagulant therapy was used to thrombogenize the pericardial effusion. After admission to the ICU, the white blood cell count was elevated and ST-segment elevation was prolonged. In anticipation of a decrease in myocardial oxygen consumption and a reduction in cardiac load, IABP support was continued and bisoprolol started. Expecting a possible cardioprotective effect, empagliflozin 10 mg and spironolactone 25 mg were started. Echocardiography showed no increase in pericardial effusion and 150 ml of additional effusion was observed until the third day. As the patient's circulation was maintained, the IABP was removed on day 15, and she was extubated on day 19. Cardiac rehabilitation was also started while taking care to prevent a re-rupture. Contrast-enhanced CT was performed on day 23, and microthrombi were observed in the peripheral pulmonary arteries. Edoxaban 15 mg was started on day 26, with careful follow-up using transthoracic echocardiography, which showed no increase in pericardial effusion. Finally, she was able to undergo cardiac Magnetic Resonance Imaging (MRI) on day 33 (Fig. 4) and walk 200 m in the ward and was transferred to the hospital for rehabilitation on day 60.

**Table 1:** Laboratory parameters after emergently transport

WBC	16.2	× 10^9^/L
RBC	4.08	× 10^12^/μL
Hb	8.3	mmol/L
Plt	241,000	/μL
TP	62	g/L
Alb	37	g/L
T-bil	24	umol/L
LDH	1064	IU/L
CK	1541	IU/L
CK-MB	56	IU/L
BUN	6.6	mmol/L
Cr	99	umol/L
Na	139	mmol/L
K	4.3	mmol/L
Cl	103	mmol/L
Ca	2.2	mmol/L
CRP	97,900	ug/L
Glu	9.2	mmol/L
Hb A1c	38.8	mmol/mol
NT-proBNP	4949	pg/mL
cTnI	75,651.4	pg/mL
pH	7.36	
PCO2	29.7	Torr
PO2	52.7	Torr
HCO3	16.7	mmol/L
BE	− 7	mmol/L
Lac	6.8	mmol/L

*WBC* White blood cell count,* RBC* Red blood cell count,* Hb* Hemoglobin,* Plt* Platelet count,* TP* Total protein,* Alb* Albumin,* T-bil* Total bilirubin,* LDH* Lactate Dehydrogenase,* CK* Creatine phosphorus kinase,* CK-MB* Creatine phosphorus kinase-MB,* BUN* Blood urea nitrogen,* Cr* Creatinine,* Na* Sodium,* K* Potassium,* Cl* Crawl,* Ca* Calcium,* CRP* C-reactive protein,* Glu* Glucose,* Hb A1c* Hemoglobin A1c,* NT-proBNP* N-Terminal pro brain natriuretic peptide,* cTnI* Cardiac troponin I,* HCO3* Bicarbonate,* BE* *Base Excess*,* Lac* Lactate

## Discussion and conclusion

Generally, the first step of treatment after diagnosis of LVFWR is pericardial drainage, IABP, intravenous transfusion and recovery to satisfactory hemodynamic status with inotropic support [5], but there are cases where pericardial drainage re-ruptures due to the release of pressure on the lesion due to increased blood pressure or effusion [6]. Accordingly, it has been reported that systolic blood pressure should not be higher than 80 mmHg [7]. Additionally, once LVFWR develops, it is generally expected to present with shock, low cardiac output syndrome, and other dramatic conditions, and maintenance of circulatory dynamics is often important, so aggressive use of IABP or percutaneous cardiopulmonary support (PCPS) is recommended [8–10]. IABP can be expected to reduce left ventricular afterload and left ventricular pressure through its systolic unloading effect, and our policy was to maintain IABP support for as long as possible [11]. As CRP elevation is reported to be one of the characteristic findings of cardiac rupture [12], the IABP placement was maintained until day 16 when this CRP turned negative while paying careful attention to the risk of thrombosis. It is generally accepted that LVFWR has a less favorable prognosis if treated without surgery [9, 13]. However, if there is a blow-out type cardiac rupture, decompression by pericardial drainage may improve left ventricular contraction and cause a sudden increase in systolic blood pressure, which may enlarge the rupture site. Assuming that bleeding becomes uncontrollable and circulatory dynamics cannot be maintained, it is necessary to make close contact with cardiovascular surgery and switch to cardiac surgery　such as sutureless repair (affixing a Tachosil: fibrinogen combined drug or pericardial patch to the rupture site), or suture. Meanwhile, considering that surgical mortality rates of 24–35% have been reported, there is no clear evidence that it should be performed in all patients [14–16]. Mathew et al. stated that some cases could be treated without surgical intervention if a large-scale study was conducted [17]. It is not rare to avoid surgical intervention for various reasons, and even if a surgical operation can be done, cases of early re-rupture after surgery have also been reported [18]. Therefore, it is very important to provide medical intervention even after surgery. In this case as well,the long-term bedrest and the administration of beta-blockers may have been important factors in preventing recurrence [19, 20]. At a heart team conference that included cardiovascular surgeons, it was determined that the patient was elderly and that surgical manipulation of the fragile myocardial tissue would result in high risk of hemostasis difficulties and transition to a blow-out type of bleeding. Therefore, we decided to delay or avoid open chest surgery to the extent possible. In the case of bleeding associated with an anterior wall infarction, it is difficult to stop bleeding due to hematoma compression. In our case of such posterior wall lesions, it is thought that the weight of the heart also contributed to hemostasis [21]. Figueras et al. have said that surgical intervention should be considered for patients that have difficulty in controlling arterial hypertension, and patients who have a recurrence of tamponade after pericardial drainage. However, “medical management might be of particular value in patients with a lateral or an inferoposterior AMI or those at very high surgical risk, such as those with a large infarct area or those > 75 years old [22].” Such patients should remain at or be transferred to an institution with surgical backup, considering the risk of re-rupture or metachronous double-rupture [23]. Moreover, even if a patient emerges out of an acute phase, there have been reports of ventricular aneurysms occurring in the chronic phase, and thus attention needs to continue thru this period [24].

We experienced a case of an elderly woman who was diagnosed with an acute exacerbation oozing-type (blow-out migration of oozing-type) left ventricular free wall rupture due to AMI. She had temporary aggravation, but subsequently was discharged from the hospital with only conservative treatment. As there are few reports on conservative treatment of left ventricular free wall rupture, we report our case as an example.

## Data Availability

Please contact the author for data request.
